# A review of radiological characteristics and patterns of fat necrosis after different autologous breast surgery techniques

**DOI:** 10.1186/s13244-026-02330-4

**Published:** 2026-06-22

**Authors:** Nieke N. P. M. Smeins, Zoë M. A. Kuijlaars, Silvia Pérez Rodrigo, Esther M. Heuts, Andrzej Piatkowski, Thiemo J. A. van Nijnatten

**Affiliations:** 1 Department of Radiology and Nuclear Medicine, Maastricht University Medical Center+, Maastricht, The Netherlands; 2 Department of Plastic, Reconstructive, and Hand Surgery, Maastricht University Medical Center+, Maastricht, The Netherlands; 3 GROW Research Institute for Oncology and Reproduction, Maastricht University, Maastricht, The Netherlands; 4 Department of Radiology, MD Anderson Cancer Center, Madrid, Spain; 5 Department of Surgery, Maastricht University Medical Center+, Maastricht, The Netherlands; 6 SHE School of Health Professions Education, Maastricht University, Maastricht, The Netherlands; 7 NUTRIM School of Nutrition and Translational Research in Metabolism, Maastricht University, Maastricht, The Netherlands; 8 Dutch Expert Center for Screening (LRCB), Nijmegen, The Netherlands

**Keywords:** Fat necrosis, Breast surgery, Ultrasonography, Mammography, Magnetic resonance imaging

## Abstract

**Background:**

The aim of this systematic review was to compare the radiological characteristics of fat necrosis distribution and morphology of tissue flaps versus autologous fat transfer (AFT) on ultrasound (US), mammography (MG), and magnetic resonance imaging (MRI), in order to improve diagnostic accuracy and guide treatment decisions.

**Materials and methods:**

This systematic review was performed according to the PRISMA guidelines. A literature search was conducted in PubMed, Embase, and Scopus databases, identifying studies published after 2005. Eligible studies included female patients undergoing breast surgery using flaps or AFT, with fat necrosis assessed on US, MG, or MRI.

**Results:**

Seventeen studies (13 AFT, 4 flap surgery) were included. Imaging was performed 1 to 240 months postoperatively. The mean prevalence of fat necrosis ranged from 12.5 to 23.6% for studies on flap surgery (*n* = 4), versus 7.3 to 82.9% for AFT (*n* = 5). On US, fat necrosis exhibited diverse echogenicity and cystic components often without vascularity, for both flap surgery and AFT. MG characteristics included radiolucent oil cysts with calcifications. MRI showed nonenhancing hypointense lesions with hyperintense borders on T1-weighted and T2-weighted fat-suppressed images. After flap surgery, fat necrosis was predominantly located at the peripheral margins of the flap, whereas in AFT it was more diffusely distributed.

**Conclusion:**

Radiological characteristics of fat necrosis are generally comparable between tissue flaps and AFT, although a difference was observed in the prevalence and distribution of fat necrosis between the two techniques. However, literature is limited, and additional research is needed to be able to refine the radiological definition of fat necrosis.

**Key Points:**

- Identifying radiological differences between fat necrosis in tissue flaps and autologous fat transfer may improve diagnosis by distinguishing typical from atypical fat necrosis presentations.
- Radiological characteristics of fat necrosis are largely comparable between flaps and autologous fat transfer, although imaging features evolve over time and distribution patterns might differ.
- Understanding radiological characteristics of fat necrosis across breast surgery techniques is essential for accurate diagnosis, guiding treatment decisions, and reducing biopsies and patient distress. Distribution patterns of fat necrosis may aid in improving diagnostic consistency.

**Graphical Abstract:**

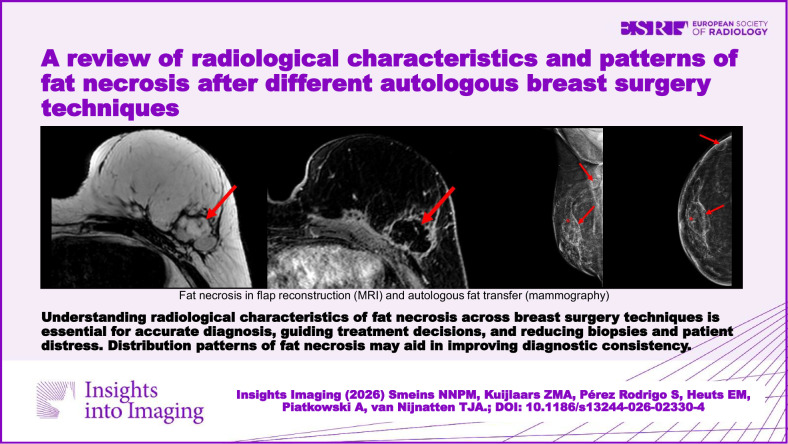

## Background

Over the last few decades, breast reconstructive surgeries have been increasingly performed following breast cancer surgery [1, 2]. Consequently, more and more imaging exams for patients who have undergone breast reconstructive surgery are requested. However, interpretation of the imaging after breast reconstruction can be challenging due to post-surgical changes [3]. Especially after autologous reconstruction, where the lack of defined margins and altered anatomy make it harder to distinguish normal tissue from recurrence [4, 5].

A diagnostically challenging aspect after autologous breast surgery is identifying fat necrosis, one of the most common postoperative findings [6]. Fat necrosis has a wide variety of presentations on imaging, which might also differ with the three phases in which fat necrosis can be divided: the acute phase (inflammation), the intermediate phase (development of oil cysts) and the late phase (fibrosis) [7, 8]. Sometimes, fat necrosis can even mimic the characteristics of (recurrent) cancer. The difficulty in differentiating between fat necrosis and cancer can lead to additional imaging, biopsies and psychological stress for the patient [9, 10].

Options for autologous breast reconstruction include tissue flaps and autologous fat transfer (AFT). In flap surgery, skin, fat, and, in specific cases, also muscle are transferred from one place of the body to the acceptor site of the breast. The most commonly used flap is the Deep Inferior Epigastric Perforator (DIEP) flap [1, 2]. Alternatively, in AFT, fat tissue is collected through liposuction, subsequently processed, and then injected into the breast site [11]. Whereas AFT was initially mainly used as an adjunct to other reconstructive techniques and for correction of contour defects, it has recently been introduced for total breast reconstruction [12, 13].

Recently, Rijkx et al [7] provided a comprehensive overview of radiological findings after total AFT breast reconstruction, describing benign and suspicious characteristics of fat necrosis. However, no studies to date have directly compared the imaging characteristics of fat necrosis between different autologous breast surgery techniques. Conceivably, the distribution and morphology of fat necrosis might vary by surgical method. Identifying such differences could improve diagnostic accuracy, enabling radiologists to more confidently distinguish typical benign fat necrosis from atypical findings that may warrant biopsy.

Therefore, the aim of this systematic review is to provide an overview of the radiological characteristics of fat necrosis distribution and morphology of tissue flaps and AFT on different imaging modalities (ultrasound (US), mammography (MG) and breast MRI) and to compare these characteristics between both surgical techniques.

## Methods

This systematic review was conducted following the Preferred Reporting Items for Systematic reviews and Meta-Analysis (PRISMA) statement [14, 15].

### Literature search

For this systematic review, articles describing radiological characteristics of fat necrosis after breast surgery were identified. The search was completed on May 21st, 2025, using the PubMed, Embase and Scopus databases. Variations on the terms “ultrasound,” “MRI,” “mammography,” “fat necrosis,” “flap,” “fat transfer,” and “breast” were used for the search. The complete literature search and used terms can be found in Appendix A.

Selection criteria were established prior to article selection. Articles were included if they involved female patients who had undergone autologous breast surgery using tissue flaps or AFT. All types of flaps and AFT techniques were eligible, also when combined with each other or with implant reconstruction. Both reconstructive and cosmetic procedures were included. In addition, characteristics of fat necrosis had to be described on US, MG or MRI. Articles had to be written in English, Spanish, French, German or Dutch. Only articles published after 2005 were included because of the improvements in imaging techniques over the years. Reviews, book chapters and case reports or original studies with less than ten participants were excluded.

Article screening was performed using Covidence (Covidence systematic review software, Veritas Health Innovation) [16], an online systematic review management tool. After automatic deduplication, titles and abstracts were screened for eligibility by two reviewers independently (Z.K. and N.S.), followed by full-text assessments based on the predefined inclusion and exclusion criteria. Discrepancies were resolved by consulting a third reviewer (T.N.).

### Data collection

Data collection was performed independently by two reviewers (Z.K. and N.S.) using a standardized Microsoft Excel (Microsoft Corporation) data extraction form [17]. Extracted data were compared to resolve discrepancies.

### Data items

The data extraction form included information on publication, study characteristics, participants, surgical intervention, radiological findings and biopsies:

- Publication details included author information, date of publishing and country.
- Study characteristics included type of study, study period, inclusion and exclusion criteria, and total follow-up time.
- Information on participants included the number of participants, number of breasts, age, BMI, smoking, breast cancer history, radiotherapy and systemic therapy.
- Surgical intervention details included indication of breast surgery (e.g., augmentation or reconstruction), surgery technique (e.g., flap or AFT), and complications. In case of flap surgery, the type of flap was noted. In case of AFT, the number of AFT sessions, injected volume and used harvesting method were noted.
- For each breast surgery method subgroup, imaging findings of fat necrosis were collected per imaging modality (US, MG, and MRI). This concerned the number of performed imaging studies, time from intervention to imaging, indication for imaging, BI-RADS score, prevalence of fat necrosis, definition/description of fat necrosis, distribution and location of fat necrosis and diameter of fat necrosis. Prevalence of fat necrosis was calculated per study, for studies in which both the total number of patients and the number of patients with fat necrosis were reported. AFT and flap surgery were assessed separately. No formal meta-analytic pooling was performed.
- Technical aspect of the different imaging modalities were also collected. For US, information on vendor, type, frequency of the transducer in MHz, and the use of Doppler techniques was collected. For MG, information on the system and projection views. For MRI, information on field strength, MR sequences and contrast administration.
- Information on biopsies included the number of biopsies performed and the histology.

When ranges are reported, these reflect the minimum and maximum of study-specific means rather than pooled summary statistics or interpretations.

### Risk of bias assessment

Risk of bias was independently assessed by Z.K. and N.S. In the occasion of no randomized controlled trials to be included, risk of bias assessment would be performed according to the Risk Of Bias In Non-randomized Studies–of Interventions (ROBINS-I) tool [18]. This tool assesses seven domains: confounding, intervention classification, participant selection, deviations from intended interventions, missing data, outcome measurement, and selective reporting. For all articles, each domain was rated from low to critical risk of bias. Discrepancies were resolved by Z.K. and N.S. through consensus.

## Results

### Study selection

**Fig. 1:**
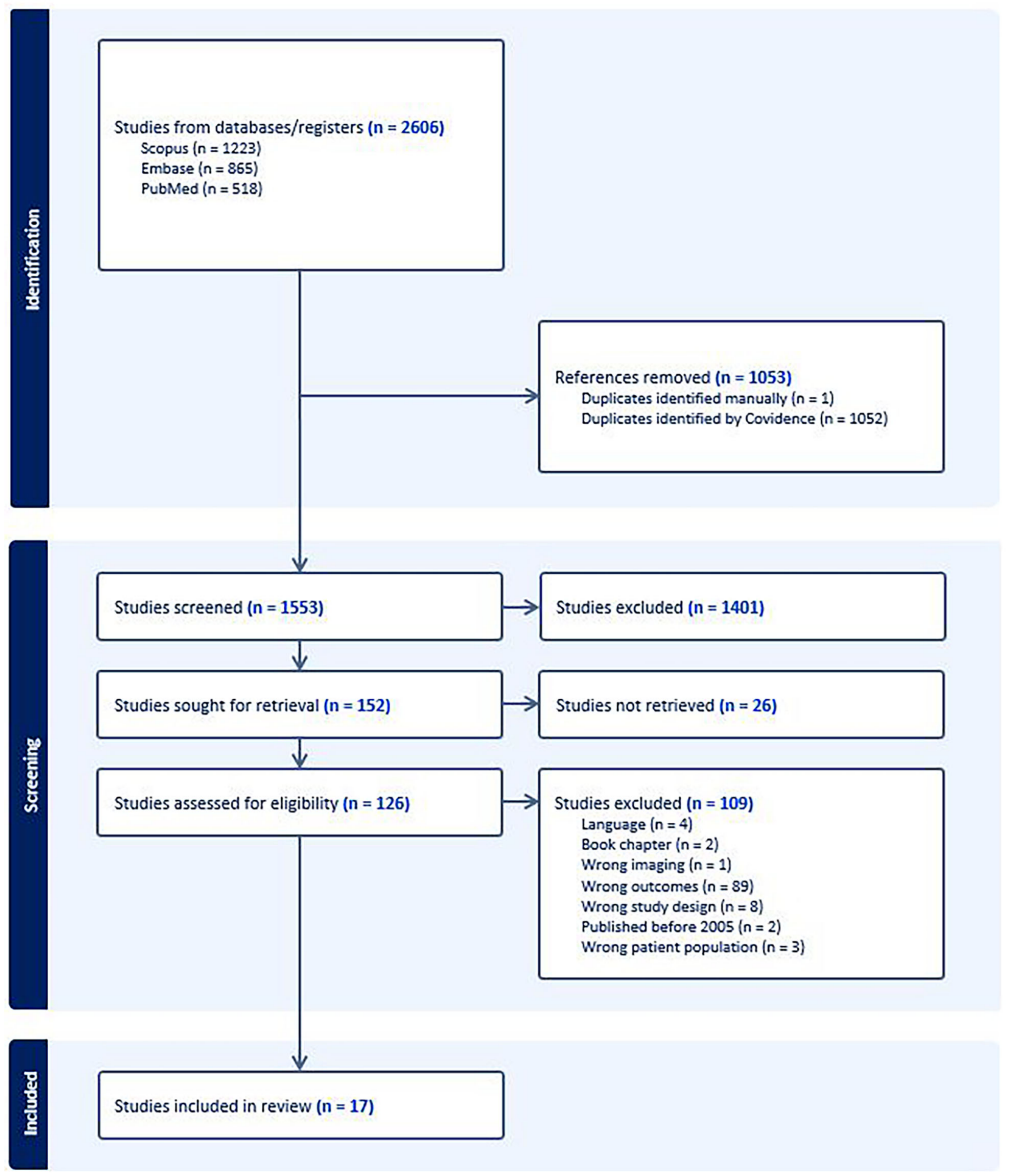
Flow-diagram of the search-strategy

The literature search retrieved 2606 potential articles from PubMed, Embase and Scopus (518, 865, and 1223, respectively). After deduplication, 1553 articles were screened on title and abstract, of which 126 articles were eligible for full-text analysis. Four articles were excluded based on language, as these were published in Chinese. After full-text analysis, seventeen articles could be included in this systematic review [19, 20]. An overview of the article selection can be found in Fig. 1.

### Study characteristics

The majority of the included studies were retrospective cohort studies (*n* = 13), and the remaining four were prospective cohort studies. None of the included studies concerned a randomized controlled trial. All studies were published between 2005 and 2023. A summary of the study characteristics is available in Table 1.

**Table 1:** General characteristics of all included studies

Study	Study design	Country	No. patients	No. breasts	Mean age ± SD (range)	Mean BMI ± SD (range)	Indication for surgery	Type of breast surgery
Kang et al [26]	R	Korea	20	NR	41 (33–46)	NR	TBR	TRAM
Kim et al [24]	R	Korea	122	130	PAP: 39.1 ± 7.3, DIEP: 47.4 ± 7.7	PAP: 22.7 ± 2.8, DIEP: 24.3 ± 3.4	TBR	DIEP or PAP flap
Moon et al [25]	R	South Korea	335	349	45.8 (27–64)	23.4 (16.1–37.3)	TBR	DIEP
Nakada et al (2018)	P	Japan	457	NR	NR	NR	BR-mix	Pedicled flap or free dermal flap
Carvajal et al [20]	P	Colombia	20	40	36.9 (31–46)	NR	CBS	AFT
Fracol et al [21]	R	United States	NR	775	49.1 ± 10.0	25.9 ± 5.3	BR-mix	AFT after flap or implant
Gosset et al [31]	R	France	21	NR	50.7 (35–64)	NR	TBR	AFT
Juhl et al [35]	P	Denmark	42	NR	53.6 ± 9.4 (33–75)	25.7 ± 3.7	PBR	AFT
Kaoutzanis et al [28]	R	United States	108	168	48 (22–71)	25.7 (19.5–38.1)	PBR	AFT
Missana et al [29]	R	France	69	74	51 (21–73)	NR	CBS	AFT
Noor et al [32]	R	United Kingdom	90	NR	53 (38–72)	NR	PBR	AFT
Parikh et al [30]	R	United States	37	69	53 (40–71)	27.4 (19.6–42.1)	BR-mix	AFT after flap or implant
Pierrefeu-Lagrange et al [33]	R	France	30	34	51 (33–63)	NR	PBR	AFT after flap
Shida et al [22]	P	Japan	256	NR	37 ± 9 (21–62)	NR	CBS	AFT
Veber et al [34]	R	France	76	NR	38.16 ± 17.3	22.49 ± 3.06	CBS	AFT
Wang et al [23]	R	China	41	82	35 ± 4 (2–51)	NR	CBS	AFT
Zheng et al [20]	R	China	66	NR	19–39	NR	CBS	AFT

*TBR* total breast reconstruction, *BR-mix* combination of partial and total breast reconstruction, *CBS* cosmetic breast surgery, *PBR* partial breast reconstruction, *R* retrospective, *P* prospective, *NR* not reported

### Patient demographics

The number of participants per study who had undergone flap surgery or AFT ranged from 20 to 457, with the exception of one study that did not specify the number of participants [21]. The mean age in the included studies ranged from 35.0 to 53.6 years and the mean BMI from 22.5 to 27.4 kg/m^2^. For the patients who received AFT, the mean age ranged from 35.0 to 53.6 years and the mean BMI from 22.5 to 27.4 kg/m^2^. For patients with flap surgery, this was 41.0–45.8 years and 23.4–23.5 kg/m^2^, respectively.

### Types of breast surgery

Among the seventeen included studies, thirteen focused on AFT and four on flap surgery. AFT was performed either for cosmetic augmentation (*n* = 5), or for reconstruction and contour deformities after breast conserving surgery or mastectomy in combination with implant and or flap reconstruction (*n* = 8). In the studies on flap surgery, patients underwent mastectomy or breast-conserving surgery prior to breast reconstruction. The techniques that were used were DIEP flap, Transverse Rectus Abdominis Myocutaneous flap (TRAM), Profunda Artery Perforator flap (PAP), pedicled flap and free dermal flap reconstruction.

### Risk of bias within studies

**Fig. 2:**
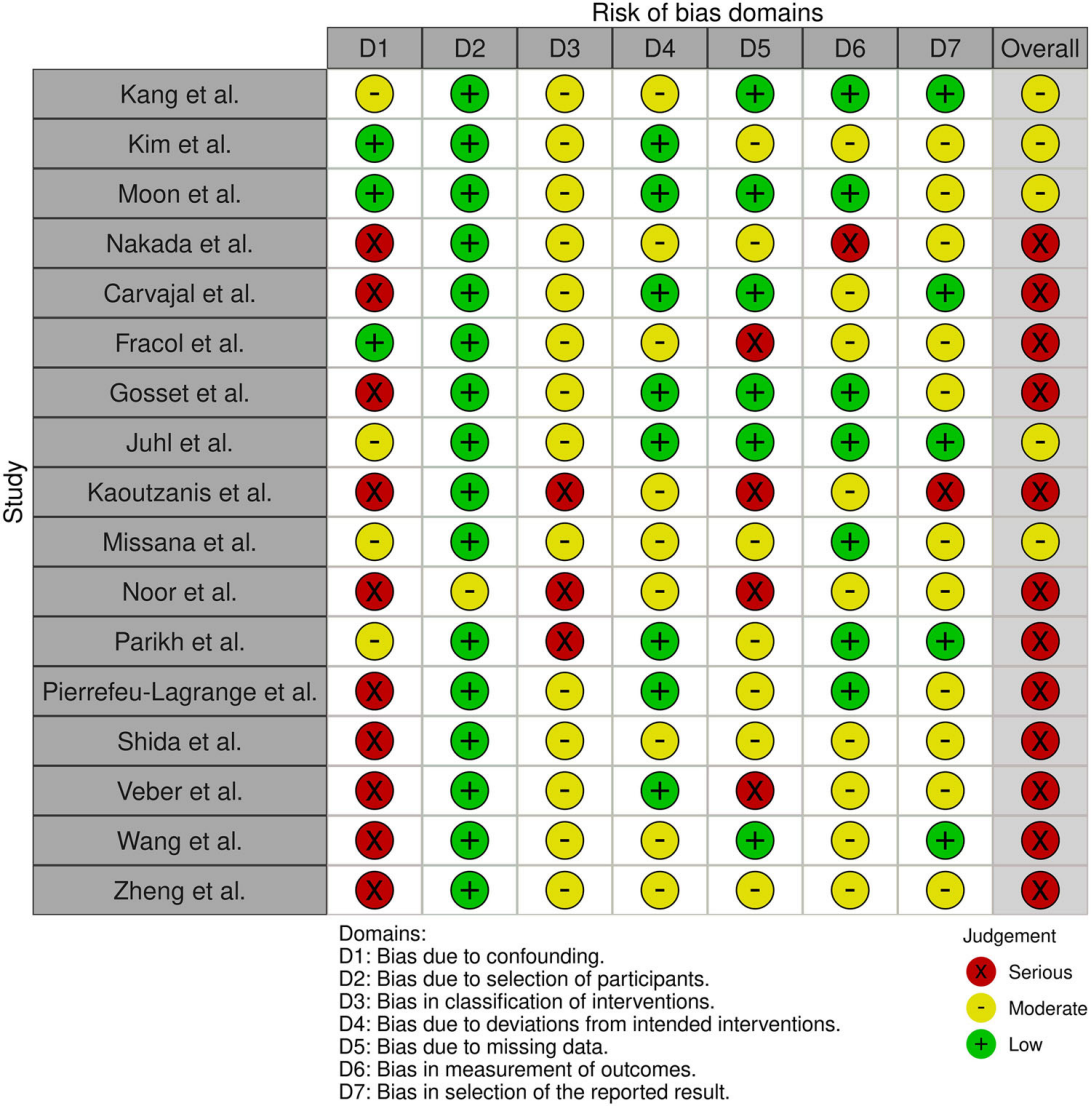
Summary of the risk of bias assessment

A summary of the risk of bias for each study is presented in Fig. 2. All studies were classified as either moderate (*n* = 5) or serious risk (*n* = 12) of bias.

### Radiographic results

#### Indications and timing for breast imaging after surgery

Seven studies implemented standardized follow-up imaging after surgery, and ten performed imaging based on clinical indications. Ten studies reported on one imaging modality, one study reported on two modalities, and six studies included all three modalities. The most frequently used imaging modality across the included studies was US (*n* = 12), followed by MG (*n* = 10) and MRI (*n* = 8). US was performed 2–240 months postoperatively, MG at 6 months–7 years, and MRI at 1–56 months. A detailed overview of imaging indications and timing per study can be found in Table 2.

**Table 2:** Indication, timing, and modalities of imaging performed

Study	Indication for breast imaging	Imaging modalities performed			Timing of the imaging
		US	MG	MRI	
Kang et al [26]	IND			✓	Mean 153 days (range 32–510 days)
Kim et al [24]	STD	✓			6 months–1 year
Moon et al [25]	IND	✓			1 year
Nakada et al (2018)	IND		✓		NR
Carvajal et al [20]	IND		✓		Mean 34.5 months (6 months–7 years)
Fracol et al [21]	IND	✓	✓	✓	NR
Gosset et al [31]	STD	✓	✓	✓	Mean 31.2 months (max 56 months)
Juhl et al [35]	STD	✓	✓		NR
Kaoutzanis et al [28]	IND	✓	✓	✓	NR
Missana et al [29]	STD			✓	Mean 11.7 months (range 1 month–3.2 years)
Noor et al [32]	IND	✓	✓	✓	Mean 11 months (range 6–15 months)
Parikh et al [30]	IND	✓			Median 9 months
Pierrefeu-Lagrange et al [33]	STD	✓	✓	✓	1 year
Shida et al [22]	IND	✓			56 months (SD 51; range 2–240 months)
Veber et al [34]	IND		✓		Median 16.2 months (SD 13.5 months)
Wang et al [23]	STD	✓			Mean 16 months (SD 7 months)
Zheng et al [20]	STD	✓	✓	✓	MG: Mean 23 months (range 12–52 months) MRI: Mean 25.4 months (range 12–53 months)

*STD* standardized imaging follow-up, *IND* imaging performed on indication, *NR* not reported

#### Technical aspects

The studies that reported technical aspects of US concerned the US system, probe and frequencies [20, 22, 23]. The studies that reported technical aspects of MG all used conventional and/or digital mammography with craniocaudal (CC) and mediolateral oblique (MLO) views. The studies that reported technical aspects of MRI all used 1.5-T systems and T1 sequences, before and after injection of gadolinium-based contrast agent, and T2 sequences. Details of the technical aspects can be found in Tables 3 and 4.

**Table 3:** Imaging findings of fat necrosis in tissue flaps

Study	Flap type	Technical details	BI-RADS	Location	Definition/appearance of fat necrosis	Timeframe
US						
Kim et al [24]	DIEP; PAP	NR	NR	NR	Ill-defined complex cystic lesions with edematous fat (subacute phase); Spiculated mass with calcified wall (late phase)	Subacute phase: days to months; Late phase: ≥ 1.5 years
Moon et al [25]	DIEP	NR	NR	Fat necrosis was most frequently detected in Holm zone III (*n* = 18, 42.9%), followed by zone II (*n* = 16, 38.1%), zone I (*n* = 3, 7.1%), and zone IV (*n* = 1, 2.4%)	Cystic, solid, or mixed lesion with/without well-defined margins, diameter ≥ 3 mm	NR
MG						
Nakada et al (2018)	Pedicled fat flap; Free dermal fat graft	NR	NR	NR	Linear coarse calcifications (Graded 0–4 based on calcifications and symptoms)	30–65 months (mean time until developing G1–G4 fat necrosis was 30 ± 8.7, 52 ± 8.2, 65 ± 6.6, and 36 ± 33 months, respectively).
MRI						
Kang et al [26]	TRAM	1.5 T Siemens/GE; T2WI, T1WI, T1FS, contrast-enhanced sequences	III-IV	Located in the contact zone between the flap and the residual breast tissue	Lesions in nonenhancing central area with irregular peripheral enhancement	Mean 153 days (range 32–510)

*NR* not reported

**Table 4:** Imaging findings of fat necrosis after AFT

Study	Technical details	BI-RADS	Location	Definition/appearance of fat necrosis	Timeframe
US					
Fracol et al [21]	NR	NR	NR	Fat necrosis: hypoechoic (48.9%) or mixed echogenicity (25.5%), circumscribed (57.4%); Oil cysts: anechoic (85.7%), circumscribed (81.0%); no irregularity or vascularity	NR
Gosset et al [31]	Siemens-Antares1 ultrasound system	NR	NR	Simple cystic images with small posterior enhancement	1 year after lipomodeling
Juhl et al [35]	Hitachi As- cendus scanner (Hitachi, Tokyo, Japan)	NR	NR	Oil cysts with a thin fibrous rim with/without calcification	Oil cyst development within 391 days; calcification development within 491 days
Kaoutzanis et al [28]	NR	NR	NR	Circumscribed hypoechoic solid mass with posterior acoustic shadowing, or lobular circumscribed mixed echotexture mass with cystic components	NR
Noor et al [32]	NR	NR	NR	Mixed fibroglandular parenchyma, scattered calcifications; well-defined lucent masses surrounded by smooth rims in the central and inner breast considered pathognomonic of fat necrosis; indeterminate calcification (circle) and 9 mm well-defined solid/cystic mass (arrow) on US	NR
Parikh et al [30]	NR	NR	Superior pole (33%), upper inner quadrant (35%), upper outer (15%), lower inner (11%), lower outer (6%)	Various types: solid masses (hypo/iso/hyperechoic), complex echogenicity, typical cystic/anechoic with posterior enhancement, cystic with internal echoes	NR
Pierrefeu-Lagrange et al [33]	Siemens-Antares ultrasound scanner	NR	NR	Simple cystic images with posterior enhancement; one case with mixed liquid/pseudosolid component; one case with a homogeneous oval hyperechoic image	NR
Shida et al [22]	HIVISION Avius with an EUP-L74M probe, Hitachi Medical Systems, Northamptonshire, UK	NR	Subcutaneous (17%), beneath mammary glands (77%), beneath pectoralis major (6%)	Cystic (oily components), complex (mixed oily/solid), solid, calcification (widespread of capsule); round anechoic shadows with posterior enhancement	NR
Wang et al [23]	Philips (Philips Medical Systems, Bothell, WA) HDI-5000 Color Doppler sonographic unit (US) with a 5 to 12 MHz linear probe	NR	Retromammary fat layer and mammary gland layer	73 cystic nodules (61.9%) anechoic with thin walls; 19 complex nodules (16.1%) with internal echoes; no blood flow on Doppler	Earliest calcification at 15 months
Zheng et al [20]	Du-5, 7e14 MHz, LOGIQ700, GE	NR	Three layers: subcutaneous (*n* = 13), glandular (*n* = 12), subglandular (*n* = 73)	Isoechoic areas at 1 week/1 month; anechoic areas at 12 months; round anechoic masses with well-defined walls	From 1 week to 12 months post-procedure
MG					
Carvajal et al [20]	Conventional and digital bilateral mammograms with craniocaudal (CC) and mediolateral oblique (MLO) views	II (85%), III (15%)	NR	Oil cysts: lucent lesions with smooth rims (calcified or not); lipid cysts with round/spherical/cluster microcalcifications	Microcalcifications as early as 11 months; oil cysts in the 2nd year; diffuse microcalcifications after 24 months
Fracol et al [21]	NR	NR	NR	NR	NR
Gosset et al [31]	GE-DMR1 mammography system with CC and oblique views	II	NR	Fatty, regular, small images with thin, regular, dense border	1 year after lipomodeling
Juhl et al [35]	Siemens Inspiration system (Siemens AG, Munich, Germany) in CC, MLO, and lateral projections.	NR	NR	Oil cysts with a thin fibrous rim with/without calcification	Oil cyst development within 391 days; calcification development within 491 days
Kaoutzanis et al [28]	NR	NR	NR	Clusters of amorphous calcifications with irregular masses or round circumscribed masses	NR
Noor et al [32]	Conventional and digital mammograms with CC and MLO projections	I-III	NR	Mixed fibroglandular parenchyma, scattered calcifications; well-defined lucent masses surrounded by smooth rims in the central and inner breast considered pathognomonic of fat necrosis	NR
Pierrefeu-Lagrange et al [33]	GE-DMR mammograph with two CC and oblique views	II	NR	Small lesions with fine, regular, dense border, sometimes with wall calcifications	NR
Veber et al [34]	NR	II	NR	Small round deposits (≤ 2 mm); cystic lesions with a thin dense cell layer and wall calcifications	NR
Zheng et al [20]	Senographe series GE Healthcare	NR	NR	Radiolucent round/ellipsoid masses with fibrous membrane; thin-walled or coarse, irregular calcifications	First seen avg. 23 months after fat grafting (range 12–52 months)
MRI					
Fracol et al [21]	NR	NR	NR	NR	NR
Gosset et al [31]	1.5 T Philips; T2, T1 FAT-SAT pre/post-gadolinium	NR	NR	Regular appearance, homogeneous hypointensity in T1 with fat suppression; not seen or in discrete hyperintensity in T2	1 year after lipomodeling
Kaoutzanis et al [28]	NR	NR	NR	NR	NR
Missana et al [29]	sagittal T1-weighted spin-echo (500/14 [repetition time, ms/echo time, ms]) and T2- weighted, fat-suppressed, fast spin-echo (4000/120). With contrast administration	NR	Upper quadrant (*n* = 1)	Circumscribed hypointense area with hyperintense rim on T1 with fat suppression; center iso/hyperintense; no enhancement after contrast	3 months after fat transfer
Noor et al [32]	NR	NR	Central and inner breast	Mixed fibroglandular parenchyma, scattered calcifications; well-defined lucent masses surrounded by smooth rims	NR
Pierrefeu-Lagrange et al [33]	1.5 T Philips; T2, T1 FAT-SAT pre/post-gadolinium	NR	NR	Homogeneous hypointense in T1 with fat suppression, difficult to see in T2; larger nodules: hypointense in T1 with hypersignal border, variable T2 appearance	NR
Zheng et al [20]	1.5 T GE; T2-weighted, T2 FSE, STIR, T1 FSE, T2 fat suppression	NR	NR	Lower signal intensity than normal fat on all sequences; signal intensity decreases on fat-suppressed sequence; calcifications visible in some large cysts	Avg. 25.4 months post-grafting (range 12–53)

*NR* not reported

#### Radiological characteristics of fat necrosis after flap surgery

US was performed in two studies at 6 to 12 months postoperatively, although it was unclear whether all patients underwent imaging. MG was reported in one study, but the timing and number of performed imaging were not specified. MRI was performed in one study on average 153 days (range 32–510) postoperatively. The mean prevalence of fat necrosis ranged from 12.5 to 23.6% within all included studies on flap surgery. The size of fat necrosis varied widely (range 0.3 to > 3 cm) [24, 25]. Fat necrosis was found most often in the peripheral portion of the flap [25] and the contact zone with the mastectomy site [26].

##### Ultrasound

Kim et al [24] observed fat necrosis as ill-defined complex cystic formations surrounded by edematous fat in the subacute phase (days to months postoperatively). Over time, these evolved into spiculated masses with calcified walls in the late phase (≥ 1.5 years postoperatively). Moon et al [25] found fat necrosis with a variable morphology, appearing as cystic, solid, or mixed formations, with or without well-defined margins.

##### Mammography

Nakada et al [27] found that fat necrosis most commonly appeared with linear, coarse calcifications, particularly notable 2–3 years after surgery. These calcifications were graded based on severity and associated symptoms, with their average time of appearance varying by grade, from approximately 30 months for grade 1 to 65 months for grade 3.

##### MRI

Kang et al [26] found fat necrosis to be characterized by nonenhancing central lesions with irregular peripheral enhancement. When fat necrosis was categorized as BI-RADS 4 on US and/or MG, the location and enhancement pattern on MRI could help in the differentiation of fat necrosis from recurrent disease.

**Fig. 3:**
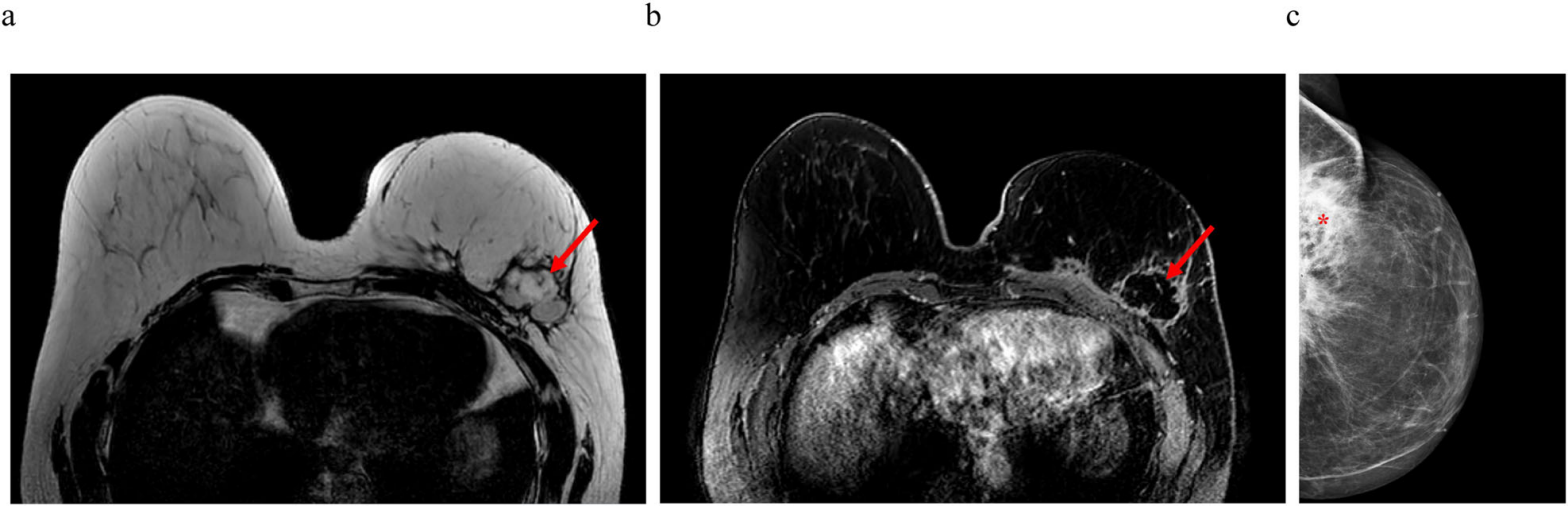
Fat necrosis after DIEP-flap breast reconstruction. A 67-year-old patient with a history of mastectomy because of DCIS underwent DIEP-flap reconstruction. Eight months after the reconstruction, fat necrosis was found in the lower outer quadrant of the left breast. MRI showed a lobed mass of 4.2 cm diameter, with surrounding tissue with mild enhancement and a specular aspect. The exam was classified BI-RADS 2 (**a**, **b**; arrow). Mammography showed coarse calcifications in the same area, probably related to the fat necrosis (**c**; asterisk)

Findings of fat necrosis after flap surgery can be found in Table 3. A case presentation of fat necrosis after DIEP-flap reconstruction following mastectomy is demonstrated in Fig. 3.

#### Radiological characteristics of fat necrosis after AFT surgery

US was performed within a range of 2 to 240 months postoperatively. MG was performed within 6 months to 7 years. MRI was performed within 1 to 56 months. The mean prevalence of fat necrosis ranged from 7.3 to 82.9%, although this information was only available in five out of thirteen AFT studies. The size of the lesions varied widely (range 5–46 mm). Fat necrosis could be located in all layers (subcutaneous, glandular, subglandular, retro- and/or intrapectoral) [22, 23, 28] and quadrants of the breast [29, 30]. The exams were most often categorized as BI-RADS 2, although BI-RADS 3 and 4 were also found [19, 30–34].

##### Ultrasound

US descriptions of fat necrosis vary widely across studies. Articles report a mix of masses with anechoic, hypoechoic, hyperechoic and mixed echogenicity and posterior enhancement or shadowing, depending on the varying stages of fat necrosis. Oil cysts are often described as a round mass with a well-defined, regular wall; however, lobular masses with cystic components are also seen. In most cases, no flow signal on Doppler is found, indicating the absence of vascularity.

##### Mammography

Fat necrosis on MG is described as small radiolucent oil cysts with smooth borders and often thin-walled calcifications. Other types of calcifications are also frequently identified. Coarse calcifications with a linear distribution were found, as well as diffuse or clustered microcalcifications. According to Carvajal et al [19], calcifications can be found as early as 11 months postoperatively. Juhl et al [35] stated that in patients in whom calcifications were found, the time between the surgery and the MG was significantly longer than in patients without calcifications.

##### MRI

Fat necrosis on MRI exams showed homogeneous hypointense lesions on T1-weighted and T2-weighted fat-suppressed images, often surrounded by a hyperintense border. After contrast administration, no enhancement was observed in fat necrosis. According to Zheng et al [20], calcifications can sometimes be found on MRI in large cysts.

**Fig. 4:**
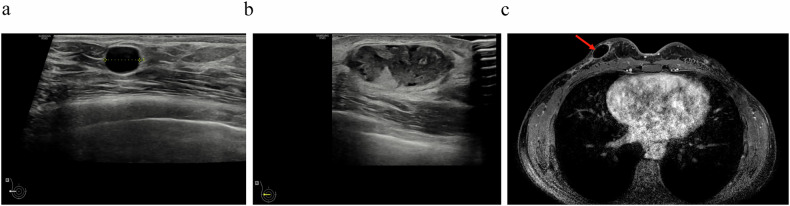
Fat necrosis after total AFT breast reconstruction. A 37-year-old patient with a history of bilateral prophylactic mastectomy, because of PALB2 gene mutation, underwent delayed total reconstruction of both breasts with AFT. After three AFT sessions, the patient presented with a painful lump in the right breast. Three palpable lumps were found during physical examination. Further diagnostics included US and MRI examination, which showed fat necrosis to be the cause. On US, the fat necrosis was described as a round mass, with mixed solid and cystic components. This was classified as BI-RADS 2 (**a**, **b**). MRI T1W-dynamic contrast-enhanced images showed a subcutaneous mass in the upper outer quadrant of her right breast, of 2.7 cm diameter with a hyperintense border (**c**; arrow)

**Fig. 5:**
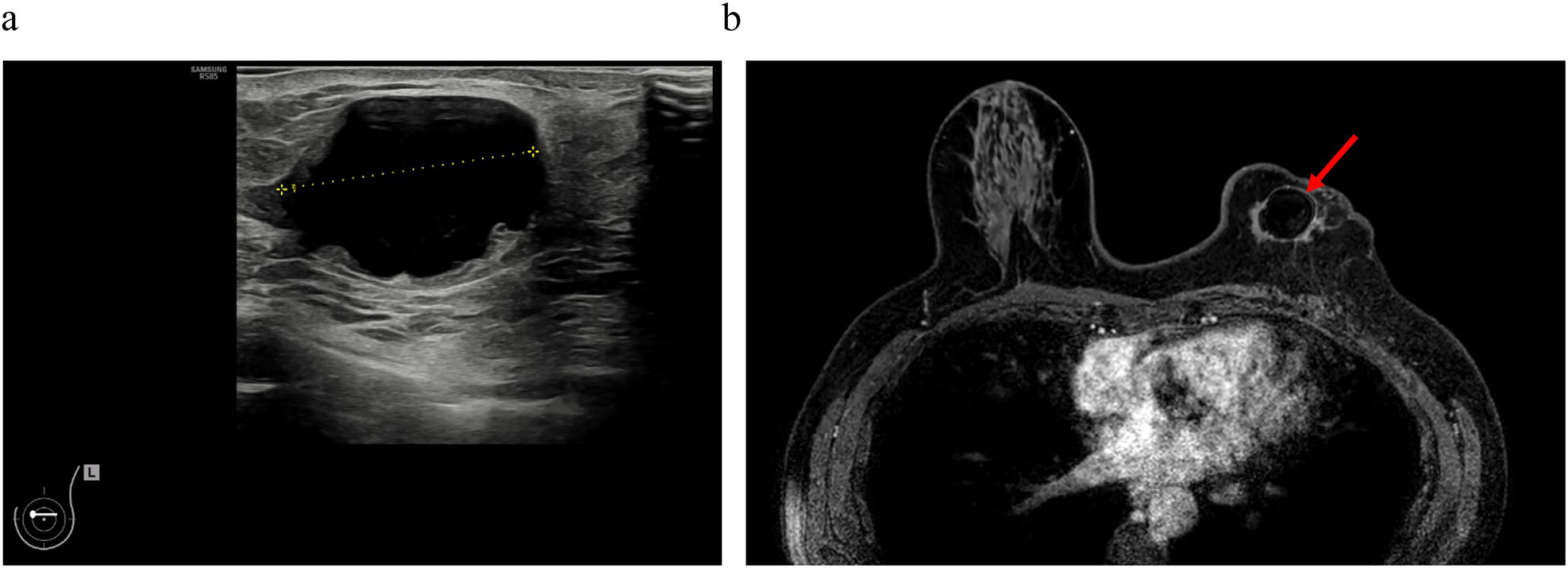
Fat necrosis after total AFT breast reconstruction. A 61-year-old patient with a unilateral mastectomy of the left breast because of DCIS, presented with a palpable lump almost 21 months after the fourth session for total AFT reconstruction. US and MRI examination showed fat necrosis. On US, multiple cysts of < 1 cm were found in the upper outer quadrant of the breast, as well as a round cyst of 2.7 cm retroareolar (**a**). MRI dynamic weighted images showed multiple oil cysts, with hyperintense borders (**b**; arrow). Both exams were classified as BI-RADS 2

**Fig. 6:**
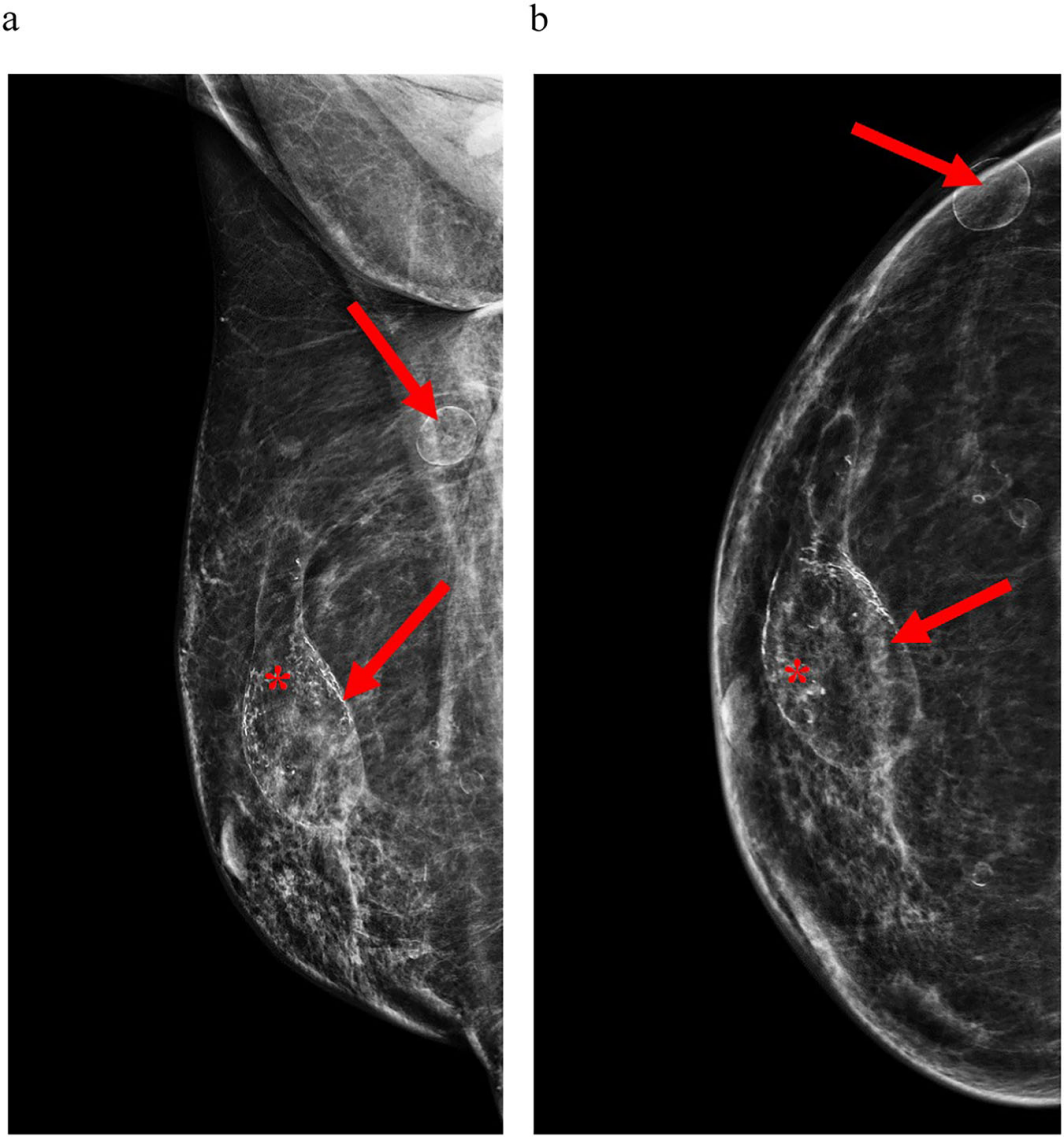
Fat necrosis after total AFT breast reconstruction. A 54-year-old patient with a history of unilateral total breast reconstruction with AFT after mastectomy of the right breast because of breast carcinoma, presented with fat necrosis on mammography 31 months after the fourth AFT session. Multiple oil cysts (arrows) were found dispersed in the right breast, as well as coarse dystrophic calcifications (asterisks). The mammography was classified BI-RADS 2 (**a** MLO, **b** CC)

Characteristics of fat necrosis after AFT surgery can be found in Table 4. Case presentations of fat necrosis after total breast reconstruction with AFT following mastectomy can be seen in Figs. 4–6.

A summary of the typical imaging features of fat necrosis in flap surgery and AFT on the different imaging modalities can be found in Table 5.

**Table 5:** Comparison of typical imaging features of fat necrosis after flap surgery and autologous fat transfer (AFT) across modalities

Imaging modality	Fat necrosis after flaps	Fat necrosis after AFT
US	– Ill-defined complex cystic lesions in (sub)acute phase, often with surrounding edematous fat. Evolve into spiculated masses with calcified walls in the late phase; – Echogenicity varies: typically hypoechoic or mixed; – Lesions are often located at the flap periphery (e.g., flap edges or contact zone); – No Doppler flow in most cases, indicating the absence of vascularity.	– Wide spectrum of appearances from anechoic simple cysts (oil cysts) with posterior enhancement to lobulated or complex cystic–solid masses. Margins are typically circumscribed (round or oval lesions with well-defined walls), though irregular margins can occur in early/inflammatory stages; – Echogenicity can be hypo-, iso-, or hyperechoic. Often a mixed pattern within the lesion; – Lesions can occur at any injection site (reported in subcutaneous, glandular, subglandular, and intramuscular layers) and in any quadrant of the breast, often multiple if fat was grafted to various areas; – No Doppler flow indicating absence of vascularity.
MG	– Fat lucencies (oil cysts) with thin calcified rims are the classic appearance. Lesions appear as radiolucent round or oval areas with smooth borders, often with peripheral rim calcifications (eggshell or coarse rim calcifications); – Calcifications in flap fat necrosis are frequently large, coarse, or “rim” calcifications, sometimes with a linear or trabecular pattern along the flap margins or the prior surgery site. These tend to develop in the late phase (typically 1–3 years post-reconstruction); – No suspicious distortion of architecture in most cases; the findings are usually clearly benign (BI-RADS 2) unless very extensive or atypical.	– Fat density cysts (oil cysts) are commonly seen, appearing as radiolucent lesions with thin fibrous rims (which may or may not calcify). Over time, rims can develop calcifications, yielding the characteristic “eggshell” calcified cyst; – Microcalcifications can also occur. Some patients develop clustered microcalcifications or amorphous calcifications in areas of fat grafting. These may appear without a distinct cyst and can mimic malignancy if in tight clusters; – Calcifications from fat necrosis have been observed as early as ~ 11 months post-AFT. Oil cysts often become evident by the second year, and more diffuse calcification patterns can manifest beyond 2 years.
MRI	– Fat necrosis in flaps typically appears as a focus of fat signal with a nonenhancing center. On T1- and T2-weighted fat-suppressed images, lesions are homogeneously hypointense (similar to fat) with a hyperintense rim representing fibrous capsule or inflammation; – No internal enhancement on post-contrast sequences (the central necrotic fat does not enhance). However, a thin peripheral rim or septal enhancement can occasionally be seen, especially in subacute cases; – Lesions are often lobulated or irregular in shape; – Predominantly located at the edges of the flap or interface with native tissue (the zone of relative ischemia).	– Fat necrosis after fat grafting appears as a localized fat-containing lesion: hypointense on fat-suppressed T1/T2 (due to macroscopic fat), often with a hyperintense rim on T1 fat-sat images. This rim corresponds to fibrous tissue or edema; it can persist into the chronic phase as a thin capsule; – Lack of enhancement: The central fatty core shows no uptake of contrast (distinguishing fat necrosis from a solid tumor). Any enhancement, if present, is usually limited to the benign rim or surrounding tissue in early-phase fat necrosis; – Distribution is diffuse/multifocal based on injection sites. Fat necrosis on MRI can be found in subcutaneous or deeper regions, wherever fat was injected, rather than one focal zone.

*US* ultrasound, *MG* mammography, *MRI* magnetic resonance imaging

#### Breast biopsies

Seven studies reported biopsies in a total of 52 patients. Fracol et al [21] reported that one of the six recurrences found in their study was initially classified as presumed fat necrosis based on US. In total, eight local recurrences were found across these studies. Although most studies did not elaborate on the details of the benign histology results, two studies confirmed most biopsies to be fat necrosis (Kaoutzanis et al [28], Parikh et al [30]). Furthermore, one study reported surgical removal of nodules for seven patients due to calcifications and patient concerns. These nodules were all histopathologically diagnosed as fat necrosis [23]. Two studies reported that no biopsies were taken [19, 20].

## Discussion

The aim of this systematic review was to investigate the radiological characteristics of the distribution and morphology of fat necrosis after autologous breast surgery. By comparing flap surgery and AFT, this study aimed to get a better understanding of the appearance of fat necrosis on different imaging modalities to be able to differentiate typical fat necrosis from atypical presentations and to possibly prevent unnecessary biopsies. In this systematic review, data from seventeen studies were included, describing radiological findings reported in the period of 2005 to 2023. The mean prevalence of fat necrosis ranged from 12.5 to 23.6% for studies on flap surgery, versus 7.3 to 82.9% for AFT. Although radiological outcomes were not the primary focus in most studies and descriptions were limited, certain patterns of fat necrosis on imaging could be identified. Overall, radiological characteristics of fat necrosis were comparable between flap surgery and AFT, with the main difference being distribution.

In the included literature, fat necrosis most often presented with benign imaging features, typically resulting in a BI-RADS 2 score [19, 27, 31–34]. However, as fat necrosis has a wide variety of presentations, differentiation from malignancies might be challenging and BI-RADS scores of 3 and 4 are not uncommon [9, 19, 23, 26, 27, 32]. BI-RADS 5, indicating a high likelihood of malignancy, should not be assigned to fat necrosis, even if it mimics suspicious features. However, when suspicious characteristics are detected, malignancy cannot be ruled out and additional diagnostics are needed. Lee et al [9] found 42 cases of fat necrosis among 362 breast reconstruction patients, of which 19% showed suspicious characteristics on imaging and needed to be pathologically confirmed. Additionally, Hembd et al [36] described 81 additional imaging studies, 42 biopsies, and 112 oncology visits in 66 patients with DIEP-flap reconstruction, all for evaluating suspicious masses or imaging abnormalities that were ultimately diagnosed as fat necrosis, stressing the severity and clinical significance of this problem. Therefore, it is essential to provide radiologists with additional tools to enhance diagnostic accuracy and prevent unnecessary biopsies.

Suspicious US findings that should raise concerns for malignancy and warrant further evaluation include heterogeneously echogenic masses with irregular, indistinct, or spiculated margins, which in rare cases exhibit vascularity. On MG, suspicious findings are often clustered microcalcifications or architectural distortion. MRI might show early-phase non-mass enhancement after contrast administration [9, 20, 37, 38]. Additional malignancy-associated features, as described by Fracol et al [21], include increased vascularity, larger lesion size, and a delayed presentation relative to benign nodules following AFT. When these imaging characteristics are present, additional imaging or biopsy should be considered, taking into account the patient’s clinical history and additional risk factors.

The optimal imaging modality to detect fat necrosis might depend on the timing of evaluation, as the presentation of fat necrosis varies over time and each modality has its advantages and limitations. Three phases can be distinguished in the natural course of fat necrosis [7, 8]. The acute phase (weeks to a few months post-surgery) consists of an inflammatory reaction in which edema can occur. Fat necrosis can be observed as an ill-defined mass or complex cystic lesion on US, often with surrounding edema. MG typically shows minimal calcification or subtle asymmetric densities, which may be occult in early stages. MRI during this phase may show focal enhancement, which should not be confused with malignancy if no solid mass is present. In the intermediate phase (several months to 1 year), fat necrosis becomes more organized, with oil cysts forming and appearing as anechoic or hypoechoic lesions on US, often with posterior enhancement. MG may reveal radiolucent cysts, and MRI shows a nonenhancing fat lesion with a fibrous rim. By this stage, any transient post-surgical enhancement usually subsides. The late phase (> 1–2 years) is characterized by the development of fibrosis. US may show a calcified mass with shadowing, or an anechoic cyst if calcification is incomplete. Fat necrosis becomes more defined with calcifications on MG, often as eggshell or coarse “popcorn” calcifications. MRI shows a hypointense fibrous scar with no enhancement.

The identified characteristics of fat necrosis and the current description of the ACR BI-RADS [39] seem to align. Fat necrosis was previously not explicitly mentioned in the ACR BI-RADS and has been incorporated since 2013 [40]. However, the current description in the ACR BI-RADS remains relatively limited and might be expanded to provide radiologists with a more comprehensive standardized definition. Especially since we observed a significant variability in the definition of fat necrosis across studies, which complicates direct comparison. Establishing a standardized definition could enhance communication among clinicians and promote more consistent and accurate diagnosis of fat necrosis, ultimately providing better guidance for treatment decisions [41].

Importantly, the reported incidence of fat necrosis might be biased due to patient selection. The studies often included patients who underwent more frequent imaging or presented with palpable lumps, potentially increasing the incidence. Additionally, as AFT is still an upcoming technique for total breast reconstruction, literature on the incidence of fat necrosis on imaging in these patients is currently lacking. However, it has been described that the risk of fat necrosis increases with larger injection volumes and more AFT sessions [42]. As larger volumes of fat are injected in total AFT reconstruction compared to partial AFT, it is reasonable to assume that more fat necrosis might be found in this group.

A difference was observed in the location and distribution of fat necrosis between AFT and flap surgery. After AFT, fat necrosis was found dispersed throughout the breast, depending on the injection sites. In contrast, following flap surgery, fat necrosis was predominantly observed at the flap margins, which may be explained by reduced perfusion in the periphery of the flap. Besides the distribution of fat necrosis, no differences could be identified between AFT and flaps. As only four included articles described fat necrosis after flap surgery, it is difficult to compare the limited results to AFT. In addition, in the included articles, AFT was often used in combination with other reconstructive techniques, such as flaps, which makes it difficult to isolate the true impact of AFT alone.

Furthermore, as fat necrosis often presents as a palpable lump, it can cause patient distress, especially in patients with a history of breast cancer. Current treatment options include surgical excision or drainage, but evidence on their effectiveness is limited. Additionally, surgical management can also lead to complications [43]. A systematic review highlighted the need for a standardized fat necrosis management algorithm and further research to determine the most effective, minimally invasive treatment strategies [44].

### Limitations

Since only a limited number of articles describe the radiological characteristics of fat necrosis, this review is based on a restricted selection of literature. Notably, more studies on fat necrosis in AFT than in flap surgery are included, which could influence the overall findings. While our systematic approach aims to minimize publication bias, the imbalance in available literature may still affect the interpretation of results. In this specific patient population, studies often focus on the clinical outcomes, rather than the radiological findings, resulting in limited quality and radiological details, as outlined in the bias assessment. Similarly, the biopsy rate and histological findings were insufficiently reported, making it unclear whether any malignancies were detected. This lack of information may have introduced potential bias.

In addition, results should be interpreted with caution as substantial heterogeneity was observed across studies, and the clinical applicability of findings depends on context-specific factors. This heterogeneity largely arises from differences in study design, as reflected by variability in imaging modalities, timing and indication of the imaging, definitions of fat necrosis, and diagnostic criteria used. Consequently, formal meta-analytic pooling was not feasible.

Furthermore, radiotherapy was not used as an exclusion criterion, despite conflicting literature suggesting that it may contribute to an increased prevalence of fat necrosis [9, 45–47]. As a result, the study population is less homogeneous, potentially leading to a higher prevalence of fat necrosis, particularly in AFT. This is relevant because AFT is more frequently performed after lumpectomy, which is almost always followed by radiotherapy, whereas larger flaps are typically used after mastectomy. However, the reporting of radiotherapy in the studies was inconsistent, further complicating the interpretation of its impact on fat necrosis prevalence.

In addition, cosmetic procedures were not excluded in this systematic review. It is plausible that the prevalence of fat necrosis differs between autologous procedures performed in healthy breast tissue and those performed after breast cancer treatment. Although this is unlikely to affect the radiological appearance of fat necrosis, it may influence its prevalence, and the results should therefore be interpreted with caution.

Many reports of fat necrosis, diagnosed either clinically or radiologically, could not be included due to insufficient radiological detail. This, in combination with the considerable heterogeneity among studies, represents an important limitation.

### Future perspectives

Future research should focus on refining the diagnostic criteria for fat necrosis, possibly by including the distribution of fat necrosis based on reconstruction technique. Furthermore, given the limited literature on total breast reconstruction using AFT, studies evaluating its long-term outcomes and incidence of fat necrosis are warranted. Finally, exploring minimally invasive treatment strategies for symptomatic fat necrosis is essential to optimize patient management while minimizing surgical interventions. Addressing these gaps in knowledge will improve diagnostic accuracy, facilitate better patient counseling, and enhance the overall management of fat necrosis in breast reconstruction.

### Educational points and recommendations

- Radiological characteristics of fat necrosis are comparable between flap surgery and AFT. However, after flap surgery, fat necrosis is most likely to be found in the peripheral margins of the flap. In contrast, after AFT, fat necrosis exhibits a more diffuse distribution.
- On US, fat necrosis can be found in all phases. However, heterogeneously echogenic masses with irregular, indistinct, or spiculated margins, often found in the acute phase, are suspect and might require additional imaging or biopsy.
- MG is particularly useful for detecting calcifications, as calcifications typically develop months to years after the initial procedure. MG has limited value in the acute phase but becomes more informative later. Fat necrosis is best identified on MG during the intermediate phase, when oil cysts are present, or in the late phase, when coarse or eggshell calcifications appear. Microcalcifications on MG are considered suspicious and usually warrant biopsy.
- MRI findings should be interpreted with caution in the acute phase, as (rim) enhancement may be present at this stage. When this is found, additional imaging or biopsy might be required to distinguish between fat necrosis and malignancy.

## Conclusion

Radiological characteristics to identify fat necrosis were comparable between flap surgery and AFT. Nevertheless, a difference was observed in the distribution of fat necrosis between the two breast surgery techniques, and certain patterns in morphology could be identified to better be able to distinguish fat necrosis from malignancy on US, MG and MRI. However, results should be interpreted with caution as literature is limited, with more studies included on AFT than on flaps, and substantial heterogeneity observed across studies. Additional research is needed to be able to refine the radiological definition of fat necrosis to promote more consistent and accurate diagnosis, ultimately providing better guidance for treatment decisions and to reduce unnecessary biopsies and patient distress. The distribution of fat necrosis based on breast surgery technique could possibly be a key factor in this.

## Supplementary information

ELECTRONIC SUPPLEMENTARY MATERIAL

## Abbreviations

AFT: Autologous fat transfer
DIEP flap: Deep inferior epigastric perforator flap
PAP flap: Profunda artery perforator flap
PRISMA statement: Preferred Reporting Items for Systematic reviews and Meta-Analysis statement
ROBINS-I tool: Risk Of Bias In Non-randomized Studies–of Interventions tool
TRAM flap: Transverse rectus abdominis myocutaneous flap
