# Supplementary material for: A review of radiological characteristics and patterns of fat necrosis after different autologous breast surgery techniques
Source: Insights Imaging. 2026 Jun 22;17:168. doi: 10.1186/s13244-026-02330-4 (PMC13287354; doi:10.1186/s13244-026-02330-4)
Supplement: Supplementary file 1 — ELECTRONIC SUPPLEMENTARY MATERIAL [file 13244_2026_2330_MOESM1_ESM.pdf]

A review of radiological characteristics and patterns of fat necrosis after different autologous breast surgery techniques

ELECTRONIC SUPPLEMENTARY MATERIAL

Appendix A. Literature search

<u>*PubMed search:*</u>

Concept	Search	Results
Imaging	("Magnetic Resonance Imaging"[Mesh] OR "Mammography"[Mesh] OR "Ultrasonography, Mammary"[Mesh] OR "Ultrasonography"[Mesh] OR "Diagnostic Imaging"[Mesh:NoExp] OR "diagnostic imaging" [Subheading] OR	
	"magnetic resonance imag*"[tiab] OR MRI[tiab] OR MRIs[tiab] OR MR[tiab] OR mammograph*[tiab] OR mastograph*[tiab] OR mammogram*[tiab] OR ultraso*[tiab] OR “ultra sound”[tiab] OR echoscop*[tiab] OR sonograph*[tiab] OR echograph*[tiab] OR echomammograph*[tiab] OR imaging[tiab] OR image[tiab] OR images[tiab])	3,454,548
	AND	
Fat necrosis	("Fat Necrosis"[Mesh] OR	
	"fat necros*"[tiab] OR “fatty necros*”[tiab] OR liponecros*[tiab] OR steatonecros*[tiab] OR cytosteatonecros*[tiab] OR adiponecros*[tiab] OR “adipose tissue necros*”[tiab] OR “oil cyst*”[tiab] OR “liponecrotic cyst*”[tiab] OR microcalcification*[tiab] OR calcification*[tiab])	78,921
	AND	
Flap	("Surgical Flaps"[Mesh] OR	
	flap[tiab] OR flaps[tiab]	
	OR	

AFT	"Adipose Tissue/transplantation"[Mesh] OR "Mammaplasty"[Mesh] OR "Plastic Surgery Procedures"[Mesh] OR	667,341
	"fat grafting"[tiab] OR "fat transfer*"[tiab] OR "fat transplantation*"[tiab] OR "fat tissue transplantation*"[tiab] OR "fat injection*"[tiab] OR lipofilling[tiab] OR lipostructuring[tiab] OR lipotransfer*[tiab] OR lipomodelling[tiab] OR AFT[tiab] OR AFG[tiab] OR reconstruct*[tiab] OR "oncoplastic surger*"[tiab] OR mammoplast*[tiab] OR mastoplast*[tiab] OR mammaplast*[tiab])	
	AND	
Breast	("Breast"[Mesh] OR	637,692
	breast[tiab] OR breasts[tiab] OR mamma[tiab] OR mammae[tiab] OR mammary[tiab])	

("Magnetic Resonance Imaging"[MeSH Terms] OR "Mammography"[MeSH Terms] OR "ultrasonography, mammary"[MeSH Terms] OR "Ultrasonography"[MeSH Terms] OR "Diagnostic Imaging"[MeSH Terms:noexp] OR "Diagnostic Imaging"[MeSH Subheading] OR "magnetic resonance imag*"[Title/Abstract] OR "MRI"[Title/Abstract] OR "MRIs"[Title/Abstract] OR "MR"[Title/Abstract] OR "mammograph*"[Title/Abstract] OR "mastograph*"[Title/Abstract] OR "mammogram*"[Title/Abstract] OR "ultraso*"[Title/Abstract] OR "ultra sound"[Title/Abstract] OR "echoscop*"[Title/Abstract] OR "sonograph*"[Title/Abstract] OR "echograph*"[Title/Abstract] OR "echomammograph*"[Title/Abstract] OR "imaging"[Title/Abstract] OR "image"[Title/Abstract] OR "images"[Title/Abstract]) AND ("Fat Necrosis"[MeSH Terms] OR "fat necros*"[Title/Abstract] OR "fatty necros*"[Title/Abstract] OR "liponecros*"[Title/Abstract] OR "steatonecros*"[Title/Abstract] OR "cytosteatonecros*"[Title/Abstract] OR "adiponecros*"[Title/Abstract] OR "adipose tissue necros*"[Title/Abstract] OR "oil cyst*"[Title/Abstract] OR "liponecrotic cyst*"[Title/Abstract] OR "microcalcification*"[Title/Abstract] OR "calcification*"[Title/Abstract]) AND ("Surgical Flaps"[MeSH Terms] OR "flap"[Title/Abstract] OR "flaps"[Title/Abstract] OR "adipose tissue/transplantation"[MeSH Terms] OR "Mammaplasty"[MeSH Terms] OR "Plastic Surgery Procedures"[MeSH Terms] OR "fat grafting"[Title/Abstract] OR "fat

transfer*"[Title/Abstract] OR "fat transplantation*"[Title/Abstract] OR "fat tissue transplantation*"[Title/Abstract] OR "fat injection*"[Title/Abstract] OR "lipofilling"[Title/Abstract] OR "lipostructuring"[Title/Abstract] OR "lipotransfer*"[Title/Abstract] OR "lipomodelling"[Title/Abstract] OR "AFT"[Title/Abstract] OR "AFG"[Title/Abstract] OR "reconstruct*"[Title/Abstract] OR "oncoplastic surger*"[Title/Abstract] OR "mammoplast*"[Title/Abstract] OR "mastoplast*"[Title/Abstract] OR "mammaplast*"[Title/Abstract]) AND ("Breast"[MeSH Terms] OR "Breast"[Title/Abstract] OR "breasts"[Title/Abstract] OR "mamma"[Title/Abstract] OR "mammae"[Title/Abstract] OR "mammary"[Title/Abstract])

21-5-2025: 518 results

Embase search:

Concept	Search	Results
Imaging	exp nuclear magnetic resonance imaging/ OR<br>exp mammography/ OR<br>exp echography/ OR<br>diagnostic imaging/ OR	
	("magnetic resonance imag*" OR MRI OR MRIs OR MR OR mammograph* OR mastograph* OR mammogram* OR ultraso* OR "ultra sound" OR echoscop* OR sonograph* OR echograph* OR echomammograph* OR imaging OR image OR images).ti,ab,kf.	4,328,451
	AND	
Fat necrosis	exp fat necrosis/ OR	
	("fat necros*" OR "fatty necros*" OR liponecros* OR steatonecros* OR cytosteatonecros* OR adiponecros* OR "adipose tissue necros*" OR "oil cyst*" OR "liponecrotic cyst*" OR microcalcification* OR calcification*).ti,ab,kf.	112,350
	AND	
Flap	exp surgical flaps/ OR<br>exp tissue flap/ OR<br>tissue graft/ or free tissue graft/ OR	
	(flap OR flaps).ti,ab,kf.	
	OR	
AFT	exp breast reconstruction/ OR	
	("fat grafting" OR "fat transfer*" OR "fat transplantation*" OR "fat tissue transplantation*" OR "fat injection*" OR lipofilling OR lipostructuring OR lipotransfer* OR lipomodelling OR AFT OR AFG OR reconstruct* OR "oncoplastic surger*" OR mammoplast* OR mastoplast* OR mammaplast*).ti,ab,kf.	596,920
	AND	
Breast	breast/ OR	
	(Breast OR Breasts OR mamma OR mammae OR mammary).ti,ab,kf.	877,522

Embase <1974 to 2025 Week 20>

1 exp nuclear magnetic resonance imaging/ or exp mammography/ or exp echography/ or diagnostic imaging/ or ("magnetic resonance imag*" or MRI or MRIs or MR or mammograph* or mastograph* or mammogram* or ultraso* or "ultra sound" or echoscop* or sonograph* or echograph* or echomammograph* or imaging or image or images).ti,ab,kf. 4328451

2 exp fat necrosis/ or ("fat necros*" or "fatty necros*" or liponecros* or steatonecros* or cytosteatonecros* or adiponecros* or "adipose tissue necros*" or "oil cyst*" or "liponecrotic cyst*" or microcalcification* or calcification*).ti,ab,kf. 112350

3 exp surgical flaps/ or exp tissue flap/ or tissue graft/ or free tissue graft/ or (flap or flaps).ti,ab,kf. 141962

4 exp breast reconstruction/ or ("fat grafting" or "fat transfer*" or "fat transplantation*" or "fat tissue transplantation*" or "fat injection*" or lipofilling or lipostructuring or lipotransfer* or lipomodelling or AFT or AFG or reconstruct* or "oncoplastic surger*" or mammoplast* or mastoplast* or mammaplast*).ti,ab,kf. 518698

5 3 or 4 596920

6 breast/ or (Breast or Breasts or mamma or mammae or mammary).ti,ab,kf. 877522

7 1 and 2 and 5 and 6 865

21-5-2025: 865 results

Scopus search:

Concept	Search	Results
Imaging	TITLE-ABS-KEY ("magnetic resonance imag*" OR MRI OR MRIs OR MR OR mammograph* OR mastograph* OR mammogram* OR ultraso* OR “ultra sound” OR echoscop* OR sonograph* OR echograph* OR echomammograph* OR imaging OR image OR images)	6,935,215
	AND	
Fat necrosis	TITLE-ABS-KEY ("fat necros*" OR “fatty necros*” OR liponecros* OR steatonecros* OR cytosteatonecros* OR adiponecros* OR “adipose tissue necros*” OR “oil cyst*” OR “liponecrotic cyst*” OR microcalcification* OR calcification*)	148,609
	AND	
Flap	TITLE-ABS-KEY (flap OR flaps)	
	OR	
AFT	TITLE-ABS-KEY ("fat grafting" OR "fat transfer*" OR "fat transplantation*" OR "fat tissue transplantation*" OR "fat injection*" OR lipofilling OR lipostructuring OR lipotransfer* OR lipomodelling OR AFT OR AFG OR reconstruct* OR "oncoplastic surger*" OR mammoplast* OR mastoplast* OR mammaplast*)	1,386,181
	AND	
Breast	TITLE-ABS-KEY (Breast OR Breasts OR mamma OR mammae OR mammary)	1,034,852

Advanced query

(TITLE-ABS-KEY (("magnetic resonance imag*" OR mri OR mris OR mr OR mammograph* OR mastograph* OR mammogram* OR ultraso* OR "ultra sound" OR echoscop* OR sonograph* OR echograph* OR echomammograph* OR imaging OR image OR images)) AND TITLE-ABS-KEY (("fat necros*" OR "fatty necros*" OR liponecros* OR steatonecros* OR cytosteatonecros* OR adiponecros* OR "adipose tissue necros*" OR "oil cyst*" OR "liponecrotic cyst*" OR microcalcification* OR calcification*)) AND TITLE-ABS-KEY ((flap OR flaps OR "fat grafting" OR "fat transfer*" OR "fat transplantation*" OR "fat tissue transplantation*" OR "fat injection*"

OR lipofilling OR lipostructuring OR lipotransfer* OR lipomodelling OR aft OR afg OR reconstruct* OR "oncoplastic surger*" OR mammoplast* OR mastoplast* OR mammaplast*)) AND TITLE-ABS-KEY ((breast OR breasts OR mamma OR mammae OR mammary)))

21-5-2025: 1,223 results
